# CXCR7 expression in diffuse large B-cell lymphoma identifies a subgroup of CXCR4+ patients with good prognosis

**DOI:** 10.1371/journal.pone.0198789

**Published:** 2018-06-19

**Authors:** María José Moreno, Alberto Gallardo, Silvana Novelli, Ana Mozos, Marc Aragó, Miguel Ángel Pavón, María Virtudes Céspedes, Víctor Pallarès, Aïda Falgàs, Miguel Alcoceba, Oscar Blanco, Marcos Gonzalez-Díaz, Jorge Sierra, Ramon Mangues, Isolda Casanova

**Affiliations:** 1 Biomedical Research Institute Sant Pau (IIB-Sant Pau), Hospital de la Santa Creu i Sant Pau, Barcelona, Spain; 2 CIBER en Bioingeniería, Biomateriales y Nanomecidicina (CIBER-BBN), Barcelona, Spain; 3 Department of Pathology, Hospital de la Santa Creu i Sant Pau, Barcelona, Spain; 4 Department of Hematology, Hospital de la Santa Creu i Sant Pau, Barcelona, Spain; 5 Department of Hematology and Pathology, IBSAL-University Hospital, Center for Cancer Research-IBMCC (USAL-CSIC), Salamanca, Spain; 6 CIBER in Oncology (CIBER-ONC), Madrid, Spain; 7 Josep Carreras Research Institute, Barcelona, Spain; Seconda Universita degli Studi di Napoli, ITALY

## Abstract

The CXCR4/CXCL12 axis has been extensively associated with different types of cancer correlating with higher aggressiveness and metastasis. In diffuse large B-cell lymphoma (DLBCL), the expression of the chemokine receptor CXCR4 is involved in the dissemination of malignant B cells and is a marker of poor prognosis. CXCR7 is a chemokine receptor that binds to the same ligand as CXCR4 and regulates de CXCR4-CXCL12 axis. These findings together with the report of CXCR7 prognostic value in several tumor types, led us to evaluate the expression of CXCR7 in diffuse large B-cell lymphoma biopsies. Here, we describe that CXCR7 receptor is an independent prognostic factor that associates with good clinical outcome. Moreover, the expression of CXCR7 associates with increased survival in CXCR4+ but not in CXCR4- DLBCL patients. Thus, the combined immunohistochemical evaluation of both CXCR7 and CXCR4 expression in DLBCL biopsies may improve their prognostic value as single markers. Finally, we show that CXCR7 overexpression in vitro is able to diminish DLBCL cell survival and increase their sensitivity to antitumor drugs. Hence, further studies on the CXCR7 receptor may establish its role in DLBCL and the molecular mechanisms that modulate CXCR4 activity.

## Introduction

Diffuse large B cell lymphoma (DLBCL) is the most frequent subtype of non-Hodgkin lymphoma, accounting for nearly 30% of all cases [1]. DLBCL is a very heterogeneous disease showing highly diverse outcomes among patients. Currently, prognosis of patients is estimated with the International Prognostic Index (IPI), which stratifies them into four risk groups [2]. However, the survival of DLBCL patients within each of the IPI groups is very heterogeneous. Thus, novel biomarkers that lead to a more accurate stratification of patients are still needed to refine the predictive scores [3].

Chemokines and their receptors play a critical role in tumorigenesis, progression and dissemination of cancer cells [4]. The CXCL12/CXCR4 axis is critical for the retention of B-cell precursors in bone marrow and homing of B lymphocytes to lymph nodes [5,6]. However, CXCR4 is not the unique receptor for CXCL12 chemokine. CXCR7 or RDC-1 was identified as a novel CXCL12 binding receptor that also binds with lower affinity to the chemokine CXCL11 [7]. CXCR7 is an atypical chemokine receptor because it is not G1-protein-coupled and does not trigger Ca2++ mobilization. CXCR7 may act as a β-arrestin-biased receptor and/or as a chemokine scavenging receptor for CXCL12 and CXCL11 [8,9]. CXCR7 is expressed in several tissues such as the hematopoietic system, heart, bone, kidney or brain. This receptor is also expressed in mature B cells and is involved in the regulation of their development and differentiation [10]. Recently, CXCR7 overexpression has been identified in several cancer types and found to be involved in the survival and growth of tumor cells [11,12]. The recent findings reporting a CXCR7-CXCL12 interaction and its implication in cancer malignancies lead to reconsider the current model established for CXCR4-CXCL12 signaling and introduce CXCR7 as a new player [13,14].

Here, we evaluate the association between CXCR7 expression and DLBCL patient survival, and if CXCR7 expression improves the prognostic value of CXCR4. We found that CXCR7 is expressed in DLBCL patients. The receptor is an independent prognostic factor that correlates with good clinical outcome. Moreover, we propose that the combined immunohistochemical evaluation of CXCR7 and CXCR4 expression in DLBCL biopsies may improve their prognostic value, as compared to their evaluation as single markers. In addition, we explore the impact of CXCR7 overexpression on proliferation and response to antitumor drugs in DLBCL cultured cells.

## Materials and methods

### Patients

Biopsies were obtained from ninety-four patients diagnosed with primary DLBCL at the Hospital de la Santa Creu i Sant Pau (HSCSP) or Hospital Universitario de Salamanca (HUS) between 2001 and 2012, based on the WHO criteria [1]. The inclusion/exclusion criteria for CXCR7 assessment have been described by our group in a previous study in which we evaluated the prognostic value of CXCR4 in the same cohort of patients [15]. Table 1 and S1 Table show the main clinical features of the patients. The Institutional Review Boards at HSCSP and HUS approved the study and the informed consent was obtained from patients according to the declaration of Helsinki. The study was performed following the REMARK guidelines [16].

**Table 1:** Association between CXCR7 expression and patients’ clinico-pathological features.

	CXCR7 expression		
Clinico-pathological features	Negative	Positive	*P*
Age			0.170
<60 *(n = 44)*	9 (.9.5)	35 (37)	
≥60 *(n = 50)*	17 (18)	33 (35)	
Gender			1
Male *(n = 50)*	14 (15)	36 (38)	
Female *(n = 44)*	12 (13)	32 (34)	
Bone marrow			1
Negative *(n = 78)*	22 (24)	56 (60)	
Positive *(n = 15)*	4 (4)	11 (12)	
Serum LDH			0.819
Normal *(n = 44)*	13 (14)	31 (33)	
High *(n = 49)*	13 (14)	36 (39)	
Stage			0.162
I-II *(n = 43)*	15 (16)	28 (31)	
III-IV *(n = 48)*	10 (11)	38 (42)	
ECOG performance status			0.751
0–2 *(n = 80)*	23 (24)	57 (61)	
> 2 *(n = 14)*	3 (3)	11 (12)	
IPI			0.052
Low risk *(n = 31)*	13 (14)	18 (20)	
Low/intermediate *(n = 24)*	5 (5.5)	19 (21)	
High/intermediate *(n = 21)*	1 (1)	20 (22)	
High risk *(n = 14)*	6 (7)	8 (9)	
Chemotherapy			1
R-CHOP *(n = 86)*	23 (25)	63 (68)	
Others *(n = 7)*	2 (2)	5 (5)	
Recurrence			0.607
No *(n = 69)*	18 (19)	51 (54)	
Yes *(n = 25)*	8 (8.5)	17 (18)	
DLBCL subtype^1^			1
Non-GCB *(n = 44)*	13 (16)	31 (38)	
GCB *(n = 37)*	11 (13.5)	26 (32)	
Condition			0.044*
Alive *(n = 75)*	17 (18)	58 (62)	
Deceased *(n = 19)*	9 (9)	10 (11)	
CXCR4			0.819
Negative *(n = 46)*	12 (13)	34 (36)	
Positive *(n = 48)*	14 (15)	34 (36)	

n (%), patients for each studied variable. P values were calculated using Fisher’s exact test.

*P value ≤ 0.05. LDH, lactate dehydrogenase; ECOG, Eastern Cooperative Group; IPI, International Prognostic Index.

^1^ Hans algorithm was used to determine the GCB/Non-GCB cases.

*P ≤ 0.05

### Immunohistochemical staining

Immunohistochemical (IHC) analysis was performed using paraffin-embedded tissue samples to assess CXCR7 expression (R&D Systems). Staining was performed in a DAKO Autostainer Link48 following the manufacturer’s instructions. IHC evaluation of CXCR4 in DLBCL patient biopsies was previously performed by our group [15]. To perform CXCR7 analysis, samples were dichotomized considering the intensity of protein expression; the DLBCL biopsies with moderate or high CXCR7 expression in at least 10% of tumor cells were considered positive, whereas the rest (less than 10% of stained cells or low intensity of expression) were considered negative. Two independent observers evaluated all samples using an Olympus BX51 microscope. There was inter-observer agreement in 95% of the samples; the remaining slides were re-evaluated and consensus decisions were made.

### Cell culture and in vitro assays

The U2932 human DLBCL cell line was obtained from DSMZ (Germany). Cells were cultured in a humidified atmosphere at 37°C in 5% CO2 with RPMI 1640 supplemented with 10% fetal bovine serum, 1% glutamine, 100 U/ml penicillin/Streptomycin (Gibco, Life Technologies). U2932 cells were transfected with the pCMV6-AC-GFP mammalian vector encoding GFP-tagged CXCR7 receptor (RG206092, Origene) or the control vector with C-terminal tGFP tag (PS100010, Origene) using the Nucleofector device (Lonza), following the manufacturer recommendations. Three days after transfection, GFP+ cells were separated using a cell sorter (BD FacAria) and cultured for 24h with complete medium. Then, cells were seeded on 96-wells plates (100μl at 2,5*10^5^ cells/ml) and cell density (number of cells/ml) and viability was quantified every 24 hours by trypan blue staining using the Countess Automated Cell Counter (Life Technologies). Antitumor activity was determined measuring cell metabolic capacity (viability) and using the Cell Proliferation Kit II (Roche Diagnostics). To that aim, cells were seeded in 96-wells plates (100μl at 2,5*10^5^ cells/ml) and exposed to vehicle or drugs, 10μM mafosfamide (Santa Cruz Biotechnology) or 1μM doxorubicin (Sigma Aldrich) for 48h. Then, cells were incubated with 50 μl of a mixture containing XTT and electron coupling reagent for 4h and absorbance was read in a spectrophotometer at 490nm (BMG Labtech). Growth inhibitory activity was obtained by subtracting the absorbance of the blanks and expressed as percentage of cell viability, as compared with untreated controls. All assays were carried out at least in triplicates.

### FACS analysis

Fluorescence-activated cell sorting (FACS) analysis was performed to detect CXCR7 expression. One million cells for each tested condition were washed in phosphate-buffered saline containing 0.5% bovine serum albumin (PBS-BSA) and fixed with 4% paraformaldehyde/2% sucrose for 10 minutes at RT. After washing again with PBS-BSA, cells were ressuspended in PBS-0,2% saponin and incubated for 45 minutes at 4°C with mouse anti-human CXCR7 APC-conjugated monoclonal antibody or mouse IgG1 APC-conjugated antibody (R&D Systems) as an isotype control. The unbound antibody was removed washing twice with PBS-BSA. Data acquisition was performed using flow cytometry (FACS Calibur, BD) and analyzed by Cell Quest Pro software. Results are expressed as mean fluorescence intensity ± standard error (SE).

### Statistical analysis

Survival rates were estimated by the Kaplan-Meier method and differences between groups were compared using the log-rank test. Progression-free survival (PFS) of DLBCL patients was calculated from the onset of treatment until relapse or death. Overall survival (OS) was calculated as the time between the onset of treatment and death or date of the last follow-up. Univariate and multivariate analyses were done using the COX proportional hazard model. Association between clinico-pathological variables and CXCR7 expression were tested using the Fisher’s exact test. In vitro experiments were performed in triplicate; values are reported as mean ± standard error. Results were analyzed using the Student’s t-test. Differences were considered significant at *p* ≤ 0.05. Statistical calculations were performed using IBM SPSS Statistics software (Release 21.0.0.0, New York, NY, USA).

## Results

### CXCR7 expression associates with increased OS in DLBCL patients

**Fig 1:**
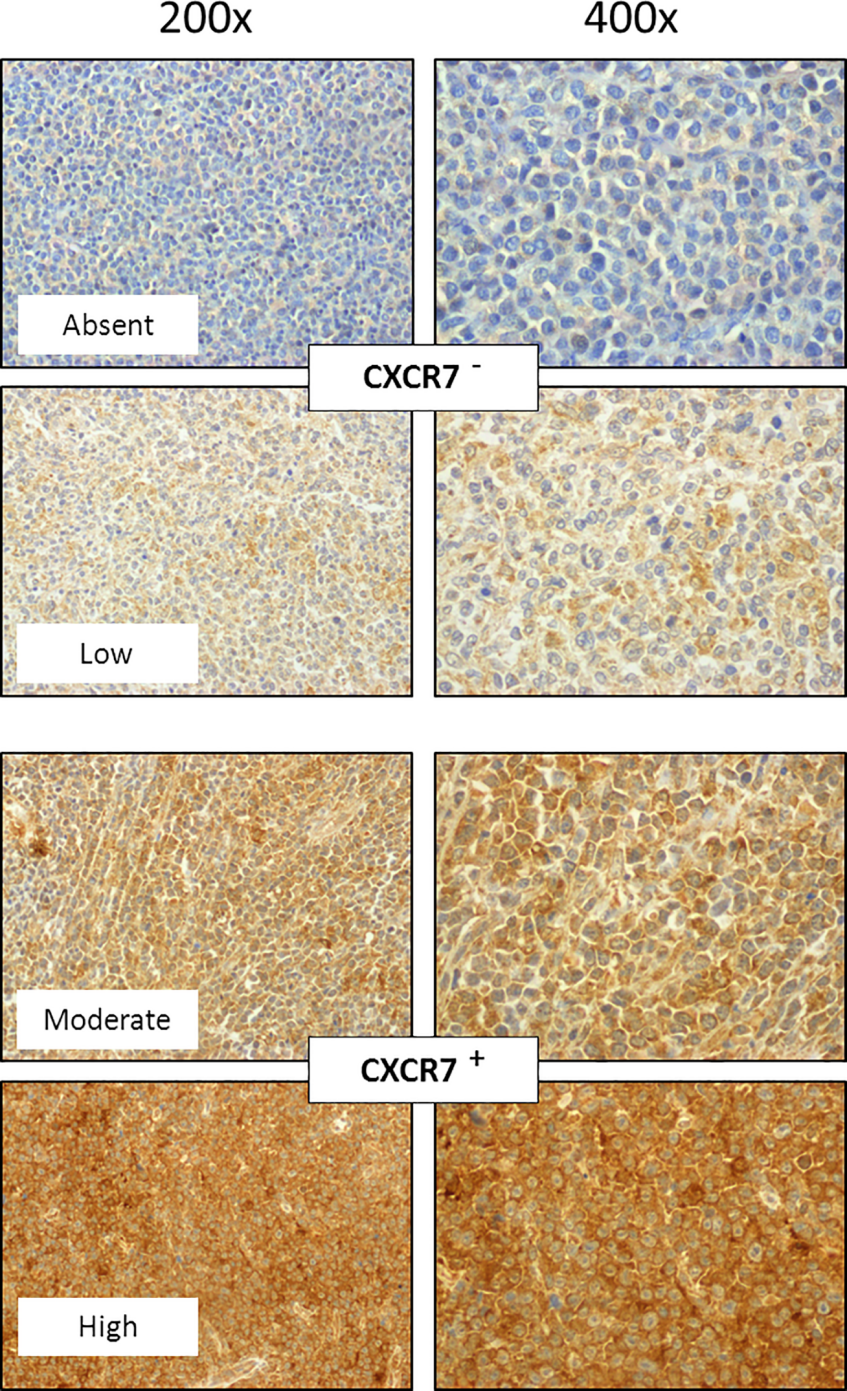
Representative CXCR7 immunostaining of DLBCL tissue sections. The DLBCL biopsies with absent or low CXCR7 expression were considered negative, whereas the biopsies with moderate or high staining were considered positive. Original magnification x200 and x400.

**Fig 2:**
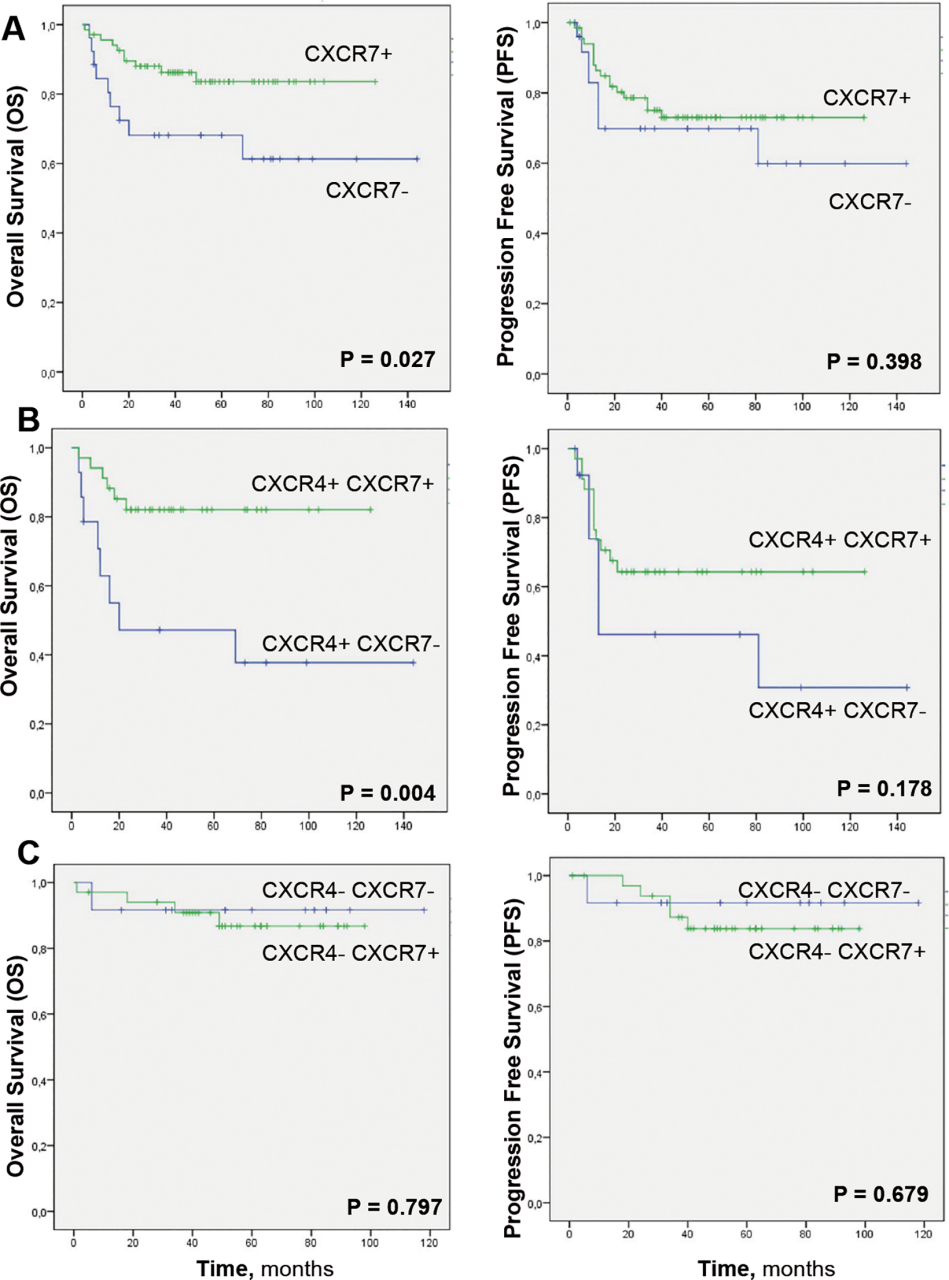
Kaplan-Meier survival analysis of DLBCL patients based on independent CXCR7 protein expression or combined CXCR7 and CXCR4 protein expression. (A) Patients bearing CXCR7+ tumors showed significantly higher OS than patients bearing CXCR7- tumors. (B) Patients bearing CXCR4+CXCR7+ tumors also showed significantly higher OS than patients bearing CXCR4+CXCR7- tumors. (C) Patients bearing CXCR4+CXCR7- tumors showed the lowest OS and PFS compared with the other patient groups, whereas patients bearing CXCR4-CXCR7+ tumors showed the highest survival.

CXCR7 immunostaining was evaluated in lymph node biopsies from patients with primary DLBCL. Representative images of samples with different levels of CXCR7 expression are shown in **Fig 1**. DLBCL biopsies with absent or low CXCR7 expression were considered negative, whereas the biopsies with moderate or high staining were considered positive. Stratification of CXCR7 expression according to the clinical features of the patients showed an association between alive patients and CXCR7-expressing tumors (**Table 1**). Moreover, Kaplan-Meier analysis using dichotomized CXCR7 values showed a significant increase in the OS, but not PFS, of patients bearing CXCR7-expressing tumors (**Fig 2A**).

### CXCR7 expression independently predicts OS in DLBCL patients

Univariate COX analysis showed that the Eastern Cooperative Oncology Group performance status (ECOG, <2 *vs* ≥2), the International Prognosis Index (IPI, 0–2 *vs* 3–4) and CXCR7 expression were significant predictors for OS (**Table 2**). Multivariate COX analysis showed that ECOG, IPI and CXCR7 remained significant prognostic factors for OS (**Table 2**). After inclusion of CXCR4 in the multivariate analysis, both CXCR4 and CXCR7 remained independent prognostic factors for OS (**Table 3**). In the univariate COX analysis for PFS, ECOG and IPI were significant predictors but CXCR7 expression was not (*P* = 0.405, not shown).

**Table 2:** Univariate and multivariate COX regression analysis for overall survival in DLBCL patients.

	Overall Survival			
	Univariate COX Regression		Multivariate COX Regression	
	HR (IC 95%)	*P*	HR (IC 95%)	*P*
**CXCR7 (+ *vs* -)**	2.668 (1.080–6.592)	0.033 *	2.764 (1.077–7.093)	0.035 *
**ECOG (0–2 *vs* >2)**	2.927 (1.112–7.706)	0.030 *	2.756 (1.020–7.448)	0.046 *
**IPI (0–2 *vs* 3–5)**	2.773 (1.072–7.173)	0.035 *	2.910 (1.099–7.709)	0.032 *
**Stage (I-II *vs* III-IV)**	1.896 (0.711–5.052)	0.201		
**LDH (normal *vs* high)**	1.004 (0.408–2.472)	0.994		
**BM (+ *vs* -)**	1.814 (0.653–5.043)	0.254		
**GCB (yes *vs* no)**	2.094 (0.645–6.805)	0.219		
**Age (< 60 *vs* ≥ 60)**	1.010 (0.410–2.487)	0.982		

Only factors identified as significant in the univariate analysis were included in the multivariate analysis. HR, hazard ratio; 95% IC, 95% confidence interval of hazard ratio; ECOG, Eastern Cooperative Group; LDH, lactate dehydrogenase; IPI, International Prognostic Index; BM, bone marrow; GCB, germinal center B-cell like.

*P ≤ 0.05

**Table 3:** Multivariate COX regression analysis for overall survival in DLBCL patients including prognostic clinical variables and both CXCR4 and CXCR7 expression.

	Multivariate COX Regression	
	HR (IC 95%)	*P*
**CXCR4 (+ *vs* -)**	3.401 (1.188–9.733)	0.023 *
**CXCR7 (+ *vs* -)**	2.605 (1.001–6.782)	0.050 *
**ECOG (0–2 *vs* >2)**	2.828 (0.996–8.029)	0.051
**IPI (0–2 *vs* 3–5)**	3.029 (1.106–8.299)	0.031 *

All the variables included in this analysis were significant factors in the univariate analysis. HR, hazard ratio; 95% IC, 95% confidence interval of hazard ratio; ECOG, Eastern Cooperative Group; IPI, International Prognostic Index.

*P ≤ 0.05

### CXCR7 expression associates with increased survival in CXCR4+ DLBCL patients

In a previous study we reported that CXCR4 overexpression correlates with cell dissemination and shorter survival in DBLCL patients [15]. Moreover, CXCR7 plays a major role in modulating CXCR4 signaling [8,9]. On this basis, we analyzed if the combined expression of CXCR4 and CXCR7 receptors improved their prognostic value.

First, we evaluated the association between CXCR7 and survival in patients bearing tumors that expressed CXCR4. The Kaplan-Meier curves showed that patients with CXCR4+CXCR7+ tumors had a significantly longer OS than patients with CXCR4+CXCR7- tumors (**Fig 2B**). Then, we performed the same analysis in patients bearing tumors that did not express CXCR4. In that case, CXCR7 had no impact on survival, since CXCR4-CXCR7+ patients showed no differences in PFS or OS with patients with CXCR4-CXC7- tumors (**Fig 2C**). These results indicate that expression of CXCR7 improve prognosis in DLBCL only when co-expressed with CXCR4, and this happens by reverting the poor prognosis associated to CXCR4+ tumors towards a favorable prognosis similar to that observed in CXCR4- tumors. Thus, CXCR7 may be responsible for blocking the aggressiveness associated with CXCR4 expressing tumors.

**Fig 3:**
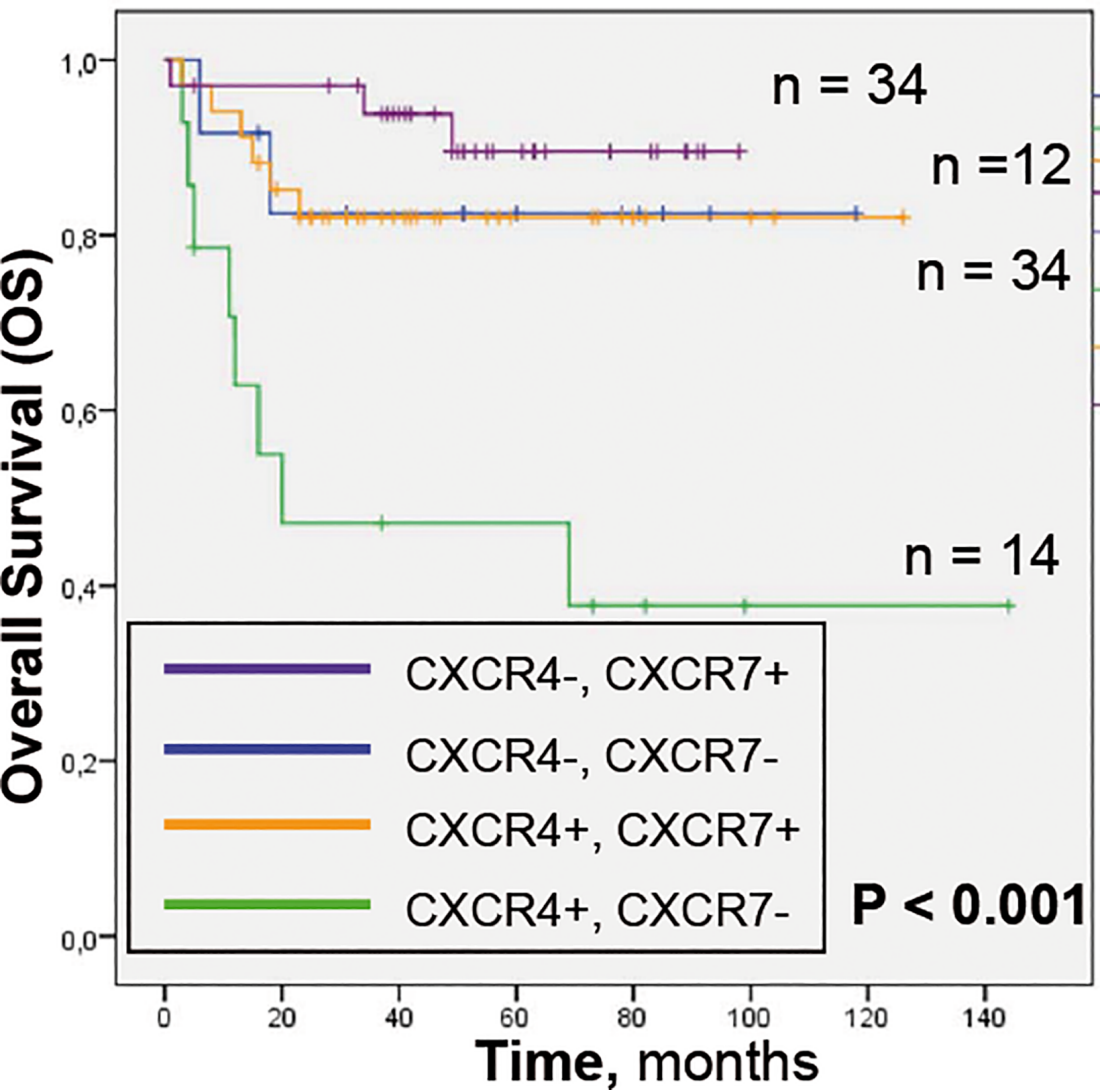
Kaplan-Meier survival analysis of DLBCL patients based on combined CXCR7 and CXCR4 protein expression. Patients bearing CXCR4+CXCR7- tumors showed the lowest OS compared with the other patient groups which had similar OS.

To confirm this observation, we established 4 different groups regarding the expression of both receptors. Kaplan-Meier curves showed that CXCR4+CXCR7+ patients had an OS similar to CXCR4- patients’ subgroups, while CXCR4+CXCR7- patients had the shortest OS (**Fig 3**). Finally, we compared the OS between patients with CXCR4+CXCR7- tumors and patients with any other combination of both receptors. CXCR4+CXCR7- expression was significantly associated with worse OS in both, univariate (p = 0,000; HR = 5,602) and multivariate COX analyses, (**Table 4**) showing higher significance than IPI or ECOG indexes.

**Table 4:** Multivariate COX regression analysis for overall survival in DLBCL patients including prognostic clinical variables, and CXCR4+CXCR7- expression versus other combinations.

	Multivariate COX Regression	
	HR (IC 95%)	*P*
**CXCR4+CXCR7- vs other combinations**	5.685 (2.099–15.398)	0.001*
**ECOG (0–2 vs >2)**	2.157 (0.742–6.2701)	0.158
**IPI (0–2 vs 3–5)**	3.102 (1.101–8.741)	0.032*

All the variables included in this analysis were significant factors in the univariate analysis. HR, hazard ratio; 95% IC, 95% confidence interval of hazard ratio; ECOG, Eastern Cooperative Group; IPI, International Prognostic Index.

*P ≤ 0.05

Thus, despite CXCR4 expression identifies DLBCL patients with poor prognosis; the patient subset expressing both CXCR7 and CXCR4 has an OS similar to that in CXCR4- patients. In turn, CXCR4+CXCR7- DLBCL patients constitute a high-risk subgroup. We suggest that the differences in patient survival may be associated with the role of CXCR7 in modulating CXCR4 signaling and aggressiveness in tumors.

### Overexpression of CXCR7 diminishes cell proliferation and increases sensitivity to antitumor drugs

**Fig 4:**
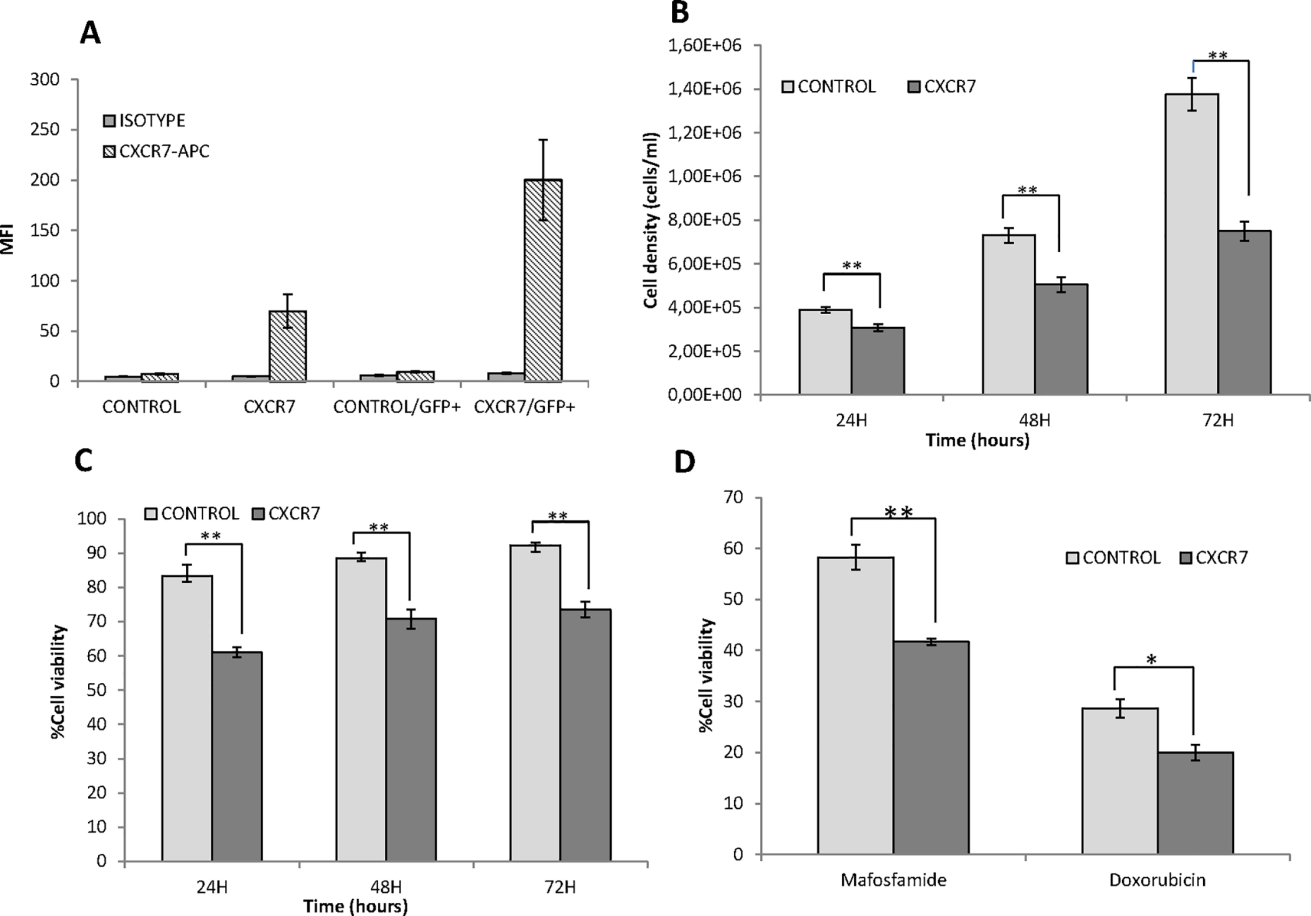
Effect of CXCR7 receptor overexpression on U2932 DLBCL cells. (A) Quantification of CXCR7 expression by flow cytometry, in the total population of transfected U2932-CXCR7 and U2932-control cells or after measurements restricted only to GFP+ cells. Values are expressed as mean fluorescence intensity (MFI) (B) Proliferation rate as measured by cell density (number of cells/ml) over time in GFP+ U2932-CXCR7 or U2932-control cells (C) Percentage of cell viability, measured by Trypan Blue staining, over time for GFP+ U2932-CXCR7 or U2932-control cells (D) Antitumor effect, expressed as percentage of cell viability respect to control cells, after 48h exposure of GFP+ U2932-CXCR7, or U2932-control cells, to 10μM mafosfamide or 1μM doxorubicin. ** p<0,01; * p<0,*05*.

In order to assess the role of CXCR7 expression in DLBCL, we transfected U2932 cells with a plasmid encoding GFP-tagged CXCR7 receptor (U2932-CXCR7). U2932 is a human DLBCL cell line showing high levels of CXCR4 expression (data not shown) and undetectable expression of CXCR7. As a control, we also transfected the same cells with an empty plasmid encoding only the GFP-tag (U2932-control). Levels of CXCR7 expression were evaluated two days after transfection in both, the total population of transfected cells (U2932-CXCR7 or U2932-control) and after the selection of GFP+ cells. As shown in **Fig 4A**, expression of the receptor was clearly increased, in the total population and in GFP+ U2932-CXCR7 cells but not in U2932-control cells. All the in vitro assays were then performed in GFP+ cells, after sorting them. We first evaluated the proliferation and cell viability of GFP+ U2932-CXCR7 and U2932-control cells over time. Interestingly, we observed that cells that overexpressed CXCR7 showed significantly lower proliferation rate and cell viability (**Fig 4B and 4C**) at all evaluated time points (24h, 48h, 72h) compared to control cells. Finally, we determined the sensitivity of cells to the antitumor compounds mafosfamide, an active analogue of cyclophosphamide, and doxorubicin: these two drugs are included in current treatment protocols for DLBCL patients. As shown in **Fig 4D**, both drugs induced higher cell death level in U2932-CXCR7 than in U2932-control cells.

## Discussion

In this study, we describe that the CXCR7 receptor is a good prognostic factor in DLBCL patients. Interestingly, this protective role is only observed in patients that overexpress CXCR4 suggesting that CXCR7 is able to overcome the higher aggressiveness induced by CXCR4.

The CXCR4/CXCL12 axis has been extensively associated with different types of cancer correlating with higher aggressiveness and metastasis [17]. CXCR4 receptor is overexpressed at least in 20 different human cancers and has become one of the most studied therapeutic targets in oncology [18]. Moreover, the role of CXCR4 in DLBCL has also been described and is related to increased dissemination of lymphoma cells and decreased survival of patients [15,19].

The discovery of CXCR7 as second receptor for CXCL12, introduced a new player in this axis deserves increasing interest [7]. CXCR7 binds CXCL12 with about ten times higher affinity than CXCR4 but it is unable to activate G protein signaling because of a modification in the DRYLAIV motif [20]. Regulation of CXCR4 by CXCR7 receptor has been reported by different, and sometimes opposite, mechanisms depending on the cell type; thus, the different tumor types could have a different regulation depending on their cell of origin.

In general, in most solid tumors, including prostate, cervical or bladder carcinomas, CXCR7 overexpression is an unfavorable prognosis marker, associated with tumor aggressiveness and metastasis [21–23]. The molecular basis of their association with aggressiveness may lay in CXCR7 ability to form heterodimers with CXCR4, acting as a signaling enhancer [24,25] and/or CXCR7 capacity for signaling on its own inducing β-arrestin-dependent ERK activation [26,27]. Thus, in tumors in which CXCR7 shows poor prognosis CXCR4 and CXCR7 may act synergistically by potentiating each other signaling.

In contrast, we have shown that CXCR7 receptor is a good prognostic factor in DLBCL patients. Importantly, CXCR7 effect on DLBCL may be dependent on CXCR4 signaling, since CXCR7 was only able to increase survival in those patients overexpressing CXCR4. Thus, patients bearing CXCR4+CXCR7+ tumors did not show differences in OS with those that were CXCR4-. Moreover, expression of CXCR7 did not show prognostic value in patients that did not express CXCR4.

In agreement with our results, an anti-tumorigenic effect has also been described for CXCR7. Liberman et al. reported the association between CXCR7 expression and favorable prognosis in neuroblastoma subtypes. They also described that CXCR7 expression reduced tumor engraftment of CXCR4+ cells in animal models and inhibited CXCR4-mediated chemotaxis [28]. Moreover, co-expression of both receptors, CXCR4 and CXCR7, has shown to inhibit the invasive properties of breast cancer cells [29]. Also in concordance with our hypothesis, D’Alterio et al. described that rectal cancer patients bearing tumors with high CXCR4 and negative/low CXCR7 expression had a poorer prognosis than patients with other marker combinations [30].

The molecular basis underlying our findings on DLBCL, as in those in other tumor types, in which CXCR7 is a marker for good prognosis may relate to a protective effect reported for CXCR7 expression, which has been always associated with the inhibition of CXCR4-mediated signaling. This may include CXCR7 ability of forming heterodimers with CXCR4 and acting as a signaling repressor [8] and/or acting as a decoy receptor able to scavenge extracellular CXCL12 leading to an inhibition of CXCR4 signal transduction [9,31]. Further experiments will be needed to address these issues.

Interestingly, and in way consistently with CXCR7 reversion of the poor outcome associated with CXCR4 expression in DLBCL and with CXCR7 acting by inhibiting CXCR4 signaling in this tumor type, in normal B-cells CXCR7 acts as an active scavenger of CXCL12 and attenuates CXCR4-mediated and CXCL12-dependent migration [32,33]. Therefore, the complex and cell-specific regulation of CXCR4 by CXCR7 may explain the different effects of CXCR7 overexpression by having a differential regulation of the CXCR4-CXL12 axis depending on the cancer type. In DLBCL as it happens in normal B-cells, CXCR7 is likely to inhibit CXCR4 signaling. This is consistent with the results obtained in the in vitro assays in cells overexpressing CXCR7 receptor. It also agrees with a previous report describing that CXCR4+ DLBCL tumors show upregulation of survival genes and downregulation of pro-apoptosis genes [19]. Thus, the lower proliferation and diminished cell viability, as well as the increased sensitivity to antitumor drugs we observed in CXCR7 overexpressing DLBCL cells support the idea that CXCR7 inhibits the cell survival signaling which is activated by CXCR4 expression. However, further studies are needed to elucidate the specific pathways that CXCR7 inhibits, and their relationship with CXCR4 signaling.

In conclusion, we show that CXCR7 expression is an independent prognostic marker associated with higher OS in DLBCL patients. The combined evaluation of CXCR4 and CXCR7 expression may improve the prediction of the clinical outcome in DLBCL. Moreover, the identification of CXCR4+CXCR7+ patients, who despite expressing CXCR4 have a good prognosis, is a finding of clinical importance. As CXCR7 expression has been reported in some hematologic malignancies, such as acute myeloid or lymphoblastic leukemia [34,35], in which CXCR4 has been described as a poor prognostic factor, it would be relevant to study if CXCR7 expression improves the CXCR4 prognostic value. Future studies are also needed to further understand the mechanism by which CXCR7 regulates the CXCR4-CXCL12 axis in DLBCL.

## Supporting information

S1 Table Clinical and pathological data of the DLBCL patients included in the analyses. (XLSX)
